# Case Report: Hepatic Artery Infusion Chemotherapy After Stage I ALPPS in a Patient With Huge HCC

**DOI:** 10.3389/fsurg.2021.746618

**Published:** 2021-11-26

**Authors:** Wenfeng Zhuo, Ang Li, Weibang Yang, Jinxin Duan, Jun Min, Jinxing Wei

**Affiliations:** Department of Hepatobiliary Surgery, Sun Yat-sen Memorial Hospital, Sun Yat-sen University, Guangzhou, China

**Keywords:** ALPPS, hepatocellular carcinoma, HAIC, FLR, hypertrophy, case report

## Abstract

Associating liver partition and portal vein ligation for staged hepatectomy (ALPPS) can induce rapid hypertrophy of the liver remnant. However, with a background of liver cirrhosis or other chronic liver diseases, patients with a huge hepatocellular carcinoma (HCC) may sometimes face insufficiency of hepatocellular regeneration after associating liver partition and portal vein ligation for staged hepatectomy (ALPPS). Herein, we report a 56-year-old male with a vast HCC (13.3 × 8.5 × 13 cm) whose ratio of the future liver remnant (FLR)/standard liver volume (SLV) was 28.7% when the disease was first diagnosed. Inadequate hypertrophy of FLR was shown in postoperative volumetric assessment a month after stage I ALPPS. After multidisciplinary team discussion (MDT), the patient was decided to follow three courses of hepatic arterial infusion chemotherapy (HAIC) with oxaliplatin, fluorouracil, and leucovorin (FOLFOX4). The last HAIC was performed together with transhepatic arterial embolization (TAE). Finally, ratio of the FLR/SLV increased from 28.7% to 40% during three-month intervals, meeting the requirements of the surgery. Stage II ALPPS, right trisectionectomy, was then successfully performed. There was no recurrence at half years of follow-up. In our case, HAIC seems to be more potent than transcatheter arterial chemoembolization (TACE) in maintaining the hyperplasia of the liver remnant, reducing tumor load, and preventing tumor progression in patients with a large HCC during ALPPS procedure. HAIC, following the first step of ALPPS, a pioneering treatment modality aiming for inadequate hypertrophy of FLR induced by ALPPS, could be an alternative procedure for patients with a vast HCC in clinical practice.

## Background

Hepatocellular carcinoma (HCC), with high incidence and mortality worldwide, has a very poor prognosis. Studies have shown that, except for liver transplantation, surgical resection is the most effective therapy for patients with HCC. However, most patients have lost the opportunity of radical resection owing to the intermediate and advanced stage of the disease when diagnosed (1). ALPPS, a novel surgical approach proposed for the deficiency of the FLR after hepatectomy, is currently mainly suitable for patients with colorectal cancer liver metastasis or with a huge HCC (> 10 cm) (2).

By inducing rapid hypertrophy of the FLR, ALPPS can strive for the chance of radical resection for patients with HCC who have lost it. However, most patients with HCC have a background of liver cirrhosis. Thus, the hypertrophy of the FLR after stage I ALPPS may be inadequate, and stage II ALPPS cannot be carried out (3).

Here, we reported a patient with a huge HCC treated in our center successfully. With insufficient hypertrophy of the FLR after stage I ALPPS, the patient was decided to follow the treatment of HAIC. The FLR then smoothly increased and finally met the requirements of surgery, so that stage II ALPPS, right trisectionectomy, can be successfully performed.

## Case Presentation

**Figure 1:**
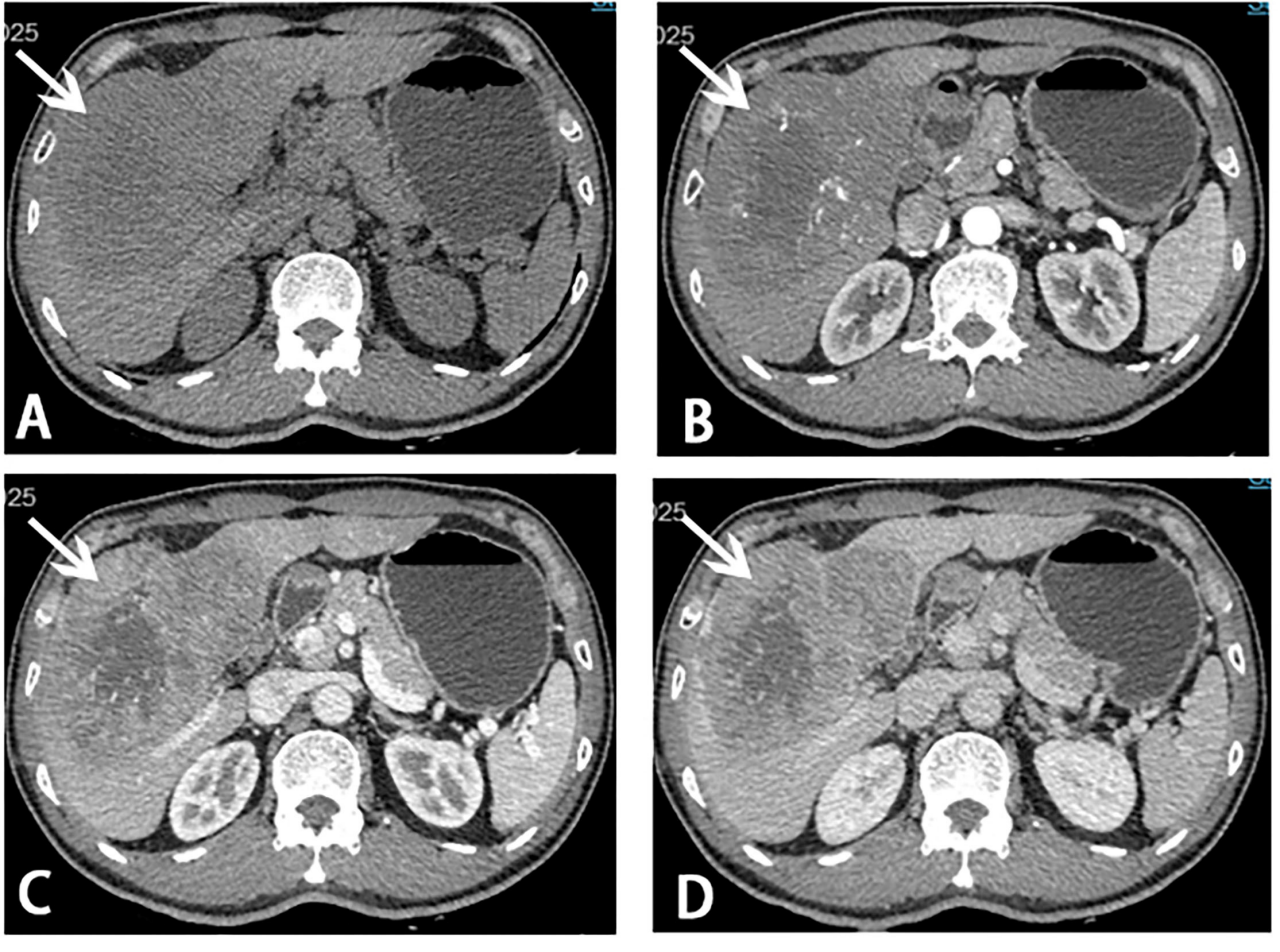
Computed tomography (CT) scans before stage I ALPPS. A giant mass (white arrow) measuring 13.3 × 8.5 × 13.0 cm in liver segments 4, 5, 6, 7, and 8 was showed on CT scans. **(A)** Plain scan; **(B)** arterial phase; **(C)** portal venous phase; **(D)** venous phase.

A 56-year-old male with the absence of clinical symptoms was diagnosed as having HCC with a huge tumor (13.3 × 8.5 × 13 cm) in liver segments 4, 5, 6, 7, and 8 (Figures 1A–D). The patient has a history of hepatitis B with mild cirrhosis but without any co-morbidities such as hypertension or diabetes. Laboratory tests indicated that liver function, blood routine examination, prothrombin time, and international normalized ratio (INR) were within the normal range. The alpha-fetoprotein (AFP) level was 231 ng/ml (normal range: 0–7 ng/ml), and the Child-Pugh score was 5. The indocyanine green retention rate at 15 min (ICG R15) was 2.7%. There was no evidence of extrahepatic metastases in a CT scan. Therefore, the patient was staged as BCLC-B according to the staging system of the Barcelona Clinic Liver Cancer (BCLC). The estimated preoperative SLV was 1,216 ml, and the FLR was 350 ml evaluated with a three-dimensional CT reconstruction system. The FLR/SLV was 28.7%, far <40%. There would be a high rate of post hepatectomy liver failure (PHLF) if radical liver resection was directly carried out (4). After an MDT, ALPPS was considered as the optimal choice to handle such a situation, and we followed it.

### Stage I ALPPS

The surgery was performed using a reverse “L” incision. Initially, the gallbladder was routinely removed. After anatomical separation, the right portal vein was identified and clipped with a hemo-lock. Liver parenchymal transection was performed with anterior approaches using an ultrasonic scalpel along the transection plane 1 cm right to the falciform ligament. During this procedure, the hepatic pedicle of liver segment 4 was identified along the right side of the sagittal part of the portal vein and was cut off. The liver was eventually cut into two parts along the above resection plane, while the liver parenchymal close to the first hepatic hilum and in front of the inferior vena cava has remained. The right hepatic pedicle could be easily observed at this time, and we signed it with dark red tape. Finally, the resection plane was covered with an absorbable gelatin sponge, and a surgical drain was routinely placed on the cut surface of liver parenchyma. The duration of surgery was 6 h, and the estimated blood loss was 150 ml. A month later, re-evaluation of FLR/SLV by CT three-dimensional reconstruction was 32%, still, <40%, which was not suitable for stage II ALPPS (4). Further interventions were needed to be carried out.

### HAIC and TAE

**Figure 2:**
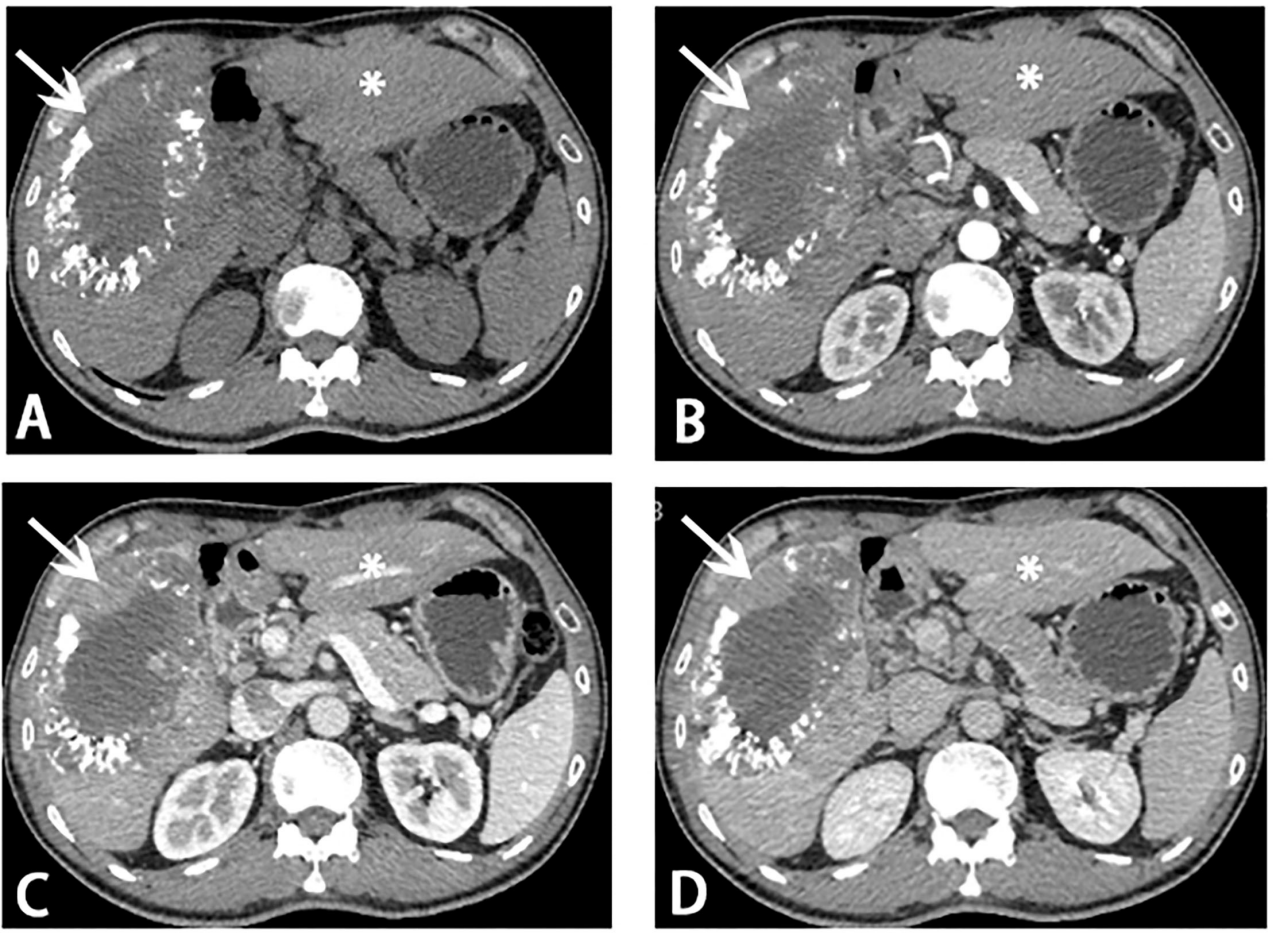
Computed tomography (CT) scans before stage II ALPPS. Reduce of tumor load (white arrow) and hypertrophy of segments 2 and 3 (asterisk) was showed on CT scans. **(A)** Plain scan; **(B)** arterial phase; **(C)** portal venous phase; **(D)** venous phase.

**Figure 3:**
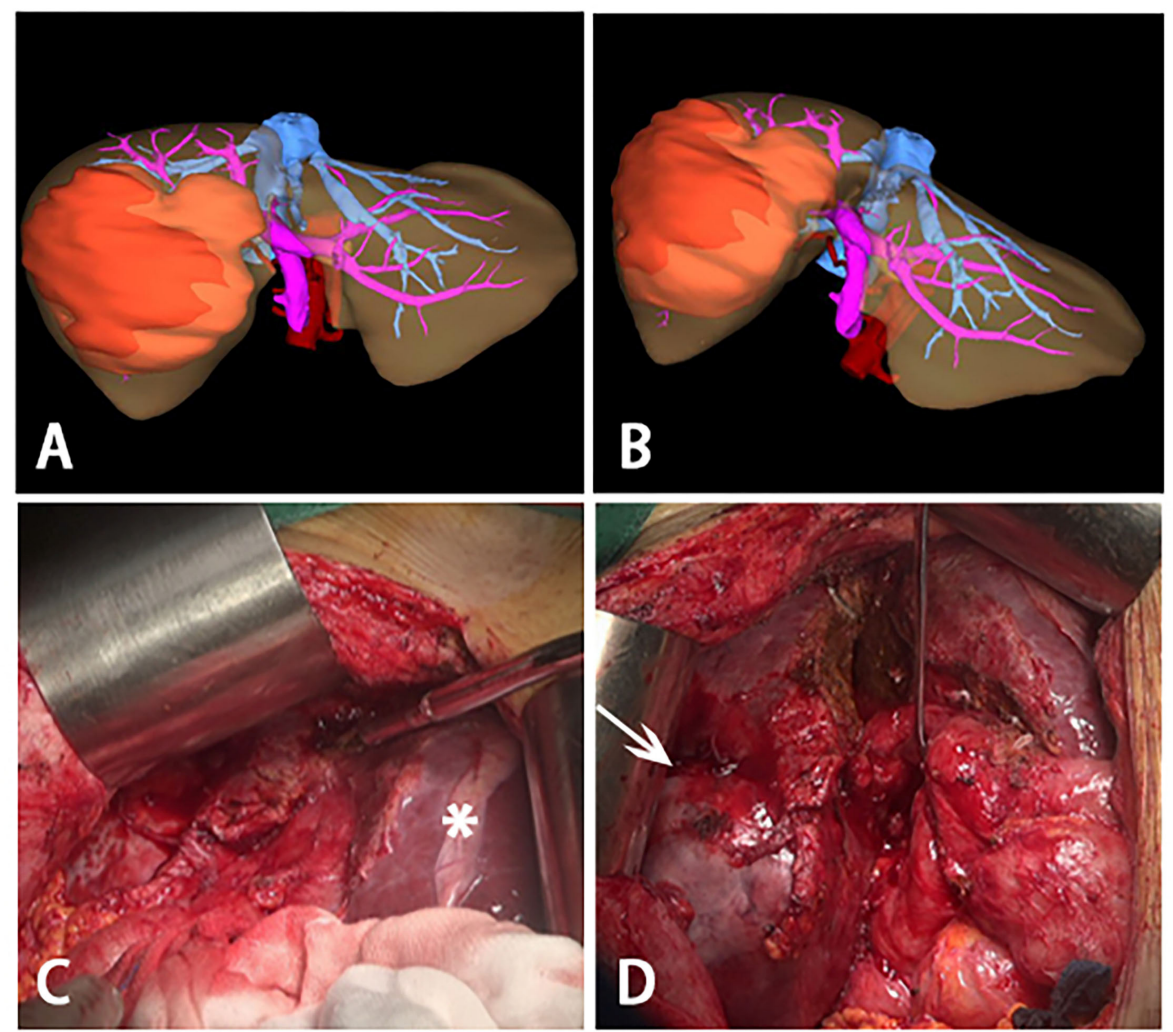
Three-dimensional reconstructions before stage II ALPPS and intraoperative photographs during stage II ALPPS. **(A,B)** Three-dimensional reconstructions before stage II ALPPS, a large tumor was on the right lobe of liver and most of the liver parenchymal between right and left was divided. **(C)** hypertrophy of the left lateral segment (asterisk). **(D)** process to remove the huge tumor (white arrow)- right trisectionectomy.

After the MDT, a new method, HAIC with FOLFOX4, was decided to be used for this patient. The patient was given three courses of HAIC therapy with a modified FOLFOX4 regimen (oxaliplatin 150 mg infusion for 2 h on day 1, leucovorin 200 mg/m^2^ infusion for 2 h on days 1 and 2, and fluorouracil 400 mg/m^2^ in bolus within 10 min on days 1 and 2, and then 2,000 mg continuous infusion for 48 h) on August 27, September 24, and November 05, 2020, respectively. The last HAIC was performed together with TAE (two arterial branches supplying the tumor were selected to completely block). After receiving about 3 months of local therapy, the follow-up imaging (Figures 2A–D) showed that the tumor load was reduced, and the FLR/SLR reached 40% (Figures 3A,B), which meets the requirements of the surgery.

### Stage II ALPPS

**Figure 4:**
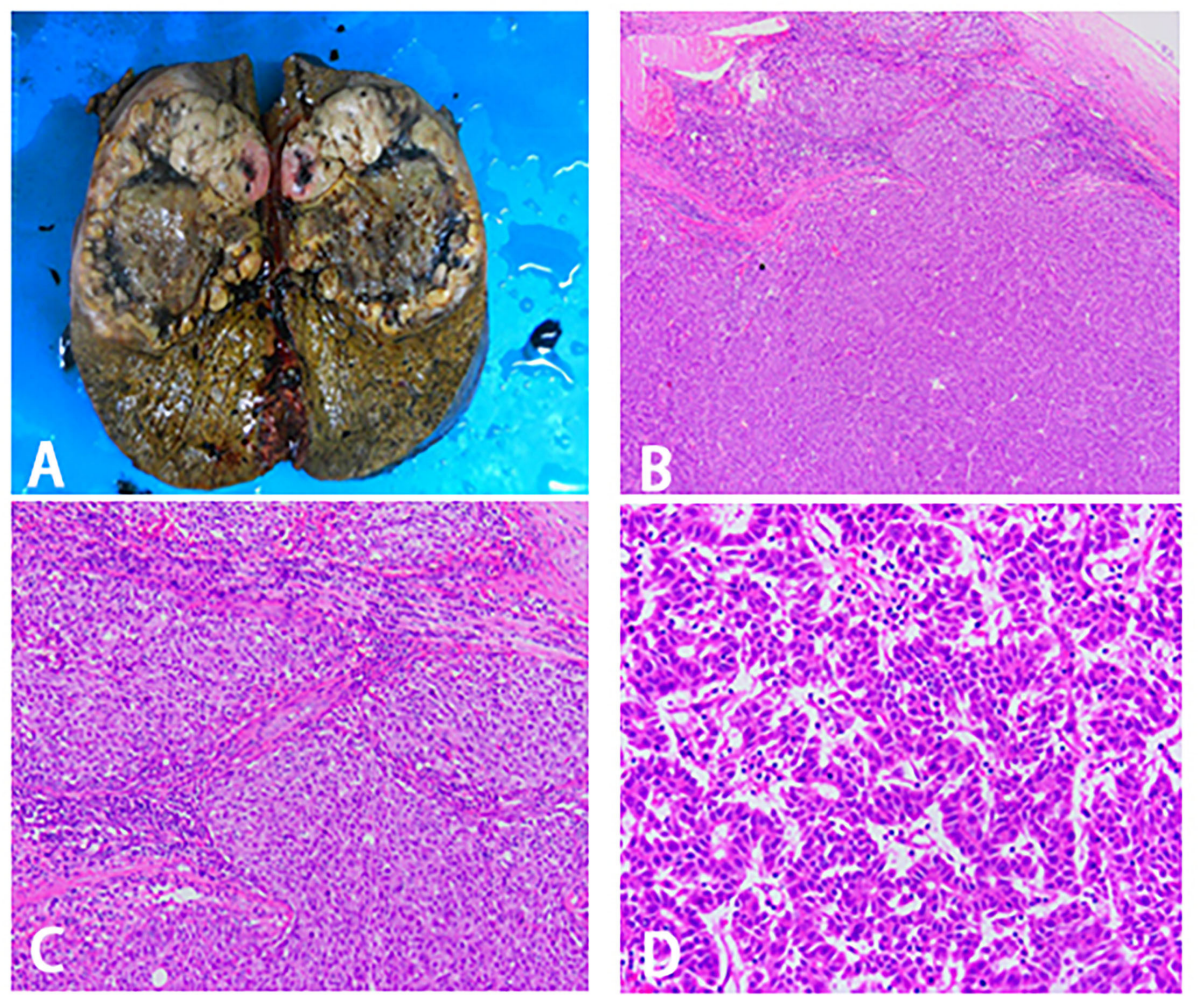
Surgical specimen and histopathological images. **(A)** Right trisectionectomy specimen. **(B–D)** Pathological examination of tissue section. **(B)** 40X magnification. **(C)** 100X magnification. **(D)** 400X magnification.

Before the operation, the ICG R15 was 4.7%, and the AFP was 8.06 ng/ml. At initial surgical exploration, hypertrophy of the left lateral segment could be easily observed (Figure 3C). The right hepatic pedicle was cut off using a linear cut stapler (LCS) after adhesion separation. Then, the remaining liver parenchyma close to the first hepatic hilum and in front of the inferior vena cava was continuously separated with an ultrasonic scalpel (Figure 3D). The right hepatic vein (RHV) and inferior right posterior vein (IRHV) were both cut off with the LCS. The right trisectionectomy was carried out until the diseased liver was totally removed. Lastly, an absorbable gelatin sponge and a surgical drain were placed over the transection plane. The operating time was 310 min, and blood loss was about 500 ml. Two units of packed red blood cells were transfused the night right after the surgery, and no eventful complications occurred during postoperative recovery. The histopathology of the tumor confirmed a 12.5 × 9 × 8.2 cm HCC in liver segments 4, 5, 6, 7, and 8. The pathology report of this tumor was moderately and poorly differentiated HCC, T1N0M0, stage IB (AJCC Cancer Staging Manual, 2018) (Figures 4A–D). There was no recurrence at half years of follow-up.

## Discussion

Hepatocellular carcinoma is one of the most common malignancies worldwide. According to studies, liver transplantation and radical hepatic resection are the only curative treatments for patients with HCC. The development of liver transplantation was severely limited because of a chronic shortage of viable donor organs (5). Hence, surgical resection is defined as the most promising treatment to improve the prognosis of patients with HCC. For patients without cirrhosis, FLR/SLR ≥ 30%, or for patients with cirrhosis, FLR/SLR ≥ 40%, if the evaluation of liver reserve function (ICG R15) is within a reasonable range, radical hepatectomy is allowed to be carried out (6). However, only 30% of patients with HCC are amenable to surgery when the disease is first diagnosed (1).

Reviewing this case, we can find that it had distinguishing characteristics as follows: 1. The size of HCC was enormous (>10 cm) when the patient was first diagnosed. 2. The patient had a background of cirrhosis. 3. FLR/SLR was far less than 40%. A high probability of PHLF would happen if radical hepatectomy was performed directly. Nowadays, for patients with a large HCC who intend to perform surgery, the mainstream modality is to conduct portal vein embolization (PVE) preoperatively to acquire adequate hypertrophy of the FLR (7). Nevertheless, it takes a long time for the hypertrophy of FLR to occur (6–8 weeks), and its success rates are not high. During this relatively long waiting time, there is a high probability of tumor progression; finally, the opportunity of radical surgery is lost (8). Although B. Romic has once pointed out a new method to treat patients with huge HCC by TACE combined with ALPPS, there is weak evidence to support it (9). Thus, given the good general health of the patient, good liver function, mild cirrhosis, and rich clinical experience of ALPPS in our center, ALPPS, which can acquire rapid hypertrophy of FLR, was selected to strive for a higher chance of curative surgery (10). Unfortunately, despite the successful completion of stage I ALPPS, postoperative evaluation still suggested insufficient FLR, as the FLR/SLV was 32%, still <40%. This low speed of hyperplasia may be related to the background of chronic liver disease (3).

A rare case has reported that after the failure of stage I ALPPS, salvage transhepatic arterial embolization (TAE) could gain the opportunity of radical hepatectomy for patients with a huge HCC (11). Considering the rich experience in HAIC of our center, HAIC, as a pioneering treatment, was selected by our center after MDT in order to keep continuous hypertrophy of FLR and prevent tumor progression. The other main reasons are listed as follows: 1. HAIC exerts maximal antitumor effect by continuous high-concentration perfusion of cytotoxic drugs while having little effect on normal liver tissue. It is safer and less likely to impair liver function and induce systemic adverse effects (12). 2. If inappropriately performed, TACE may cause necrosis of huge HCC and even normal liver tissue, resulting in acute complications with life-threatening risks. 3. Acute hypoxia of the liver can be caused by TACE and may lead to revascularization and local recurrence of the tumor (13). 4. A new randomized controlled trial on HAIC has reported that HAIC shows better efficacy than TACE in patients with HCC larger than 7 cm (14). 5. HAIC has been developed in Asian countries for many years, and it seems that HCC yields a better objective response rate with HAIC among Asians (15). To sum up, HAIC was naturally selected by us after stage I ALPPS in this case. Once the requirements were met, stage II ALPPS was encouragingly carried out.

The key feature of our case was the first usage of HAIC worldwide after stage I ALPPS with insufficient hypertrophy of the FLR for a patient with HCC. It seems that we have gotten a good outcome. Similar cases have never been reported before; so, we want to share with readers a novel method for treating patients with a colossal HCC.

## Conclusions

A new procedure, HAIC with FOLFOX4, could be an alternative treatment in case of insufficient hypertrophy of the FLR after the first step of ALPPS. It is expected to win more opportunities for curative liver resection for patients with a huge HCC. We try to name the procedure as ALPPS -HAIC. However, few studies are conducted in this area. More studies are needed to confirm the safety, feasibility, and efficacy of our method.

## Data Availability Statement

The original contributions presented in the study are included in the article/supplementary material, further inquiries can be directed to the corresponding author/s.

## Ethics Statement

Written informed consent was obtained from the individual(s) for the publication of any potentially identifiable images or data included in this article.

## Author Contributions

JM and JW performed the surgery. WZ designed the study and wrote the original draft. AL revised the manuscript. WY and JD collected and arranged imaging and pathological data. All the authors contributed to the article and approved the submitted version.

## Funding

The National Natural Science Foundation of China, and its grant numbers is 81702406.

## Conflict of Interest

The authors declare that the research was conducted in the absence of any commercial or financial relationships that could be construed as a potential conflict of interest.

## Publisher's Note

All claims expressed in this article are solely those of the authors and do not necessarily represent those of their affiliated organizations, or those of the publisher, the editors and the reviewers. Any product that may be evaluated in this article, or claim that may be made by its manufacturer, is not guaranteed or endorsed by the publisher.
